# The immunomodulatory role of matrix metalloproteinases in colitis-associated cancer

**DOI:** 10.3389/fimmu.2022.1093990

**Published:** 2023-01-19

**Authors:** Luying He, Qianming Kang, Ka Iong Chan, Yang Zhang, Zhangfeng Zhong, Wen Tan

**Affiliations:** ^1^ School of Pharmacy, Lanzhou University, Lanzhou, China; ^2^ Macao Centre for Research and Development in Chinese Medicine, Institute of Chinese Medical Sciences, University of Macau, Macao, Macao SAR, China

**Keywords:** matrix metalloproteinases (MMPs), colitis associated cancer (CAC), immunomodulation, inflammation, extracellular matrix (ECM)

## Abstract

Matrix metalloproteinases (MMPs) are an important class of enzymes in the body that function through the extracellular matrix (ECM). They are involved in diverse pathophysiological processes, such as tumor invasion and metastasis, cardiovascular diseases, arthritis, periodontal disease, osteogenesis imperfecta, and diseases of the central nervous system. MMPs participate in the occurrence and development of numerous cancers and are closely related to immunity. In the present study, we review the immunomodulatory role of MMPs in colitis-associated cancer (CAC) and discuss relevant clinical applications. We analyze more than 300 pharmacological studies retrieved from PubMed and the Web of Science, related to MMPs, cancer, colitis, CAC, and immunomodulation. Key MMPs that interfere with pathological processes in CAC such as MMP-2, MMP-3, MMP-7, MMP-9, MMP-10, MMP-12, and MMP-13, as well as their corresponding mechanisms are elaborated. MMPs are involved in cell proliferation, cell differentiation, angiogenesis, ECM remodeling, and the inflammatory response in CAC. They also affect the immune system by modulating differentiation and immune activity of immune cells, recruitment of macrophages, and recruitment of neutrophils. Herein we describe the immunomodulatory role of MMPs in CAC to facilitate treatment of this special type of colon cancer, which is preceded by detectable inflammatory bowel disease in clinical populations.

## 1 Introduction

### 1.1 Classification and structural characteristics of matrix metalloproteinases

**Figure 1:**
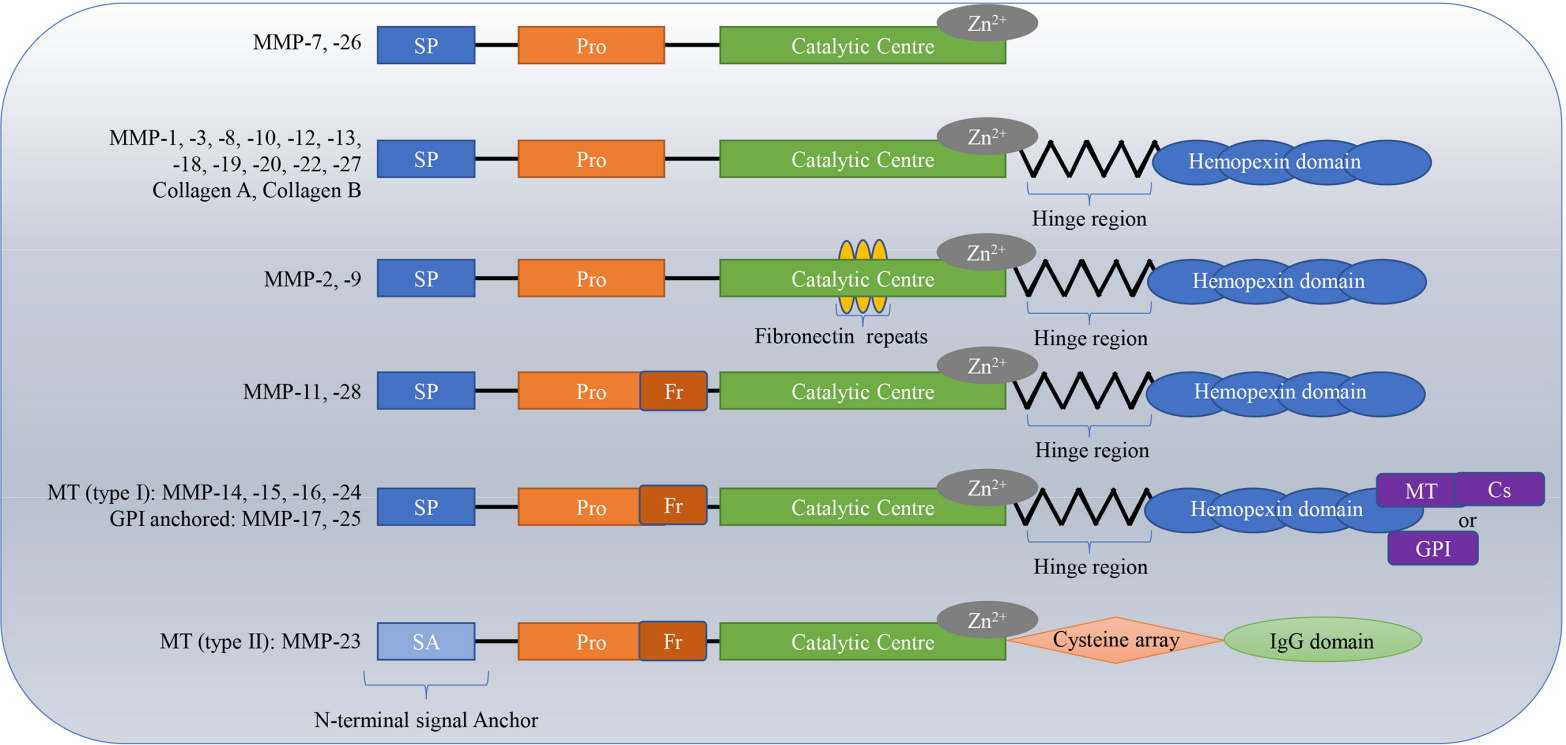
Classifications and structures of matrix metalloproteinases (MMPs). The homologous domains of these MMPs include the signal peptide domain (SP), propeptide domain (Pro), catalytic domain, and hinge region or linker peptide along with a hemopexin domain. The hinge region connects the catalytic domain to the hemopexin domain. Matrilysins lack a C-terminal hemopexin-like domain linked by a hinge or linker region; Gelatinases have special additional fibronectin repeats in the catalytic domain; MT-MMPs have a C-terminal transmembrane domain with a short cytoplasmic tail. MT, membrane-type; Cs, cytosolic; GPI, glycosylphosphatidylinositol.

MMPs are a kind of calcium-and zinc-dependent proteolytic enzyme (1), that exist in invertebrates, vertebrates and plants (2). They are produced by multiple cells and tissues, with neutrophils and dermal fibroblasts being the main sources (3). Connective tissue, pro-inflammatory and uteroplacental cells, including endothelial cells, osteoblasts, cytotrophoblasts, lymphocytes, macrophages, and vascular smooth muscle are also capable of secreting MMPs (4). Degrading the ECM is the main function of MMPs (5). The ECM plays an important role in the proliferation, growth, organization, differentiation, migration of cells, and in the exchange among information cells; it also acts as a physical barrier for microorganisms (6, 7). To date, 28 types of MMPs have been found. The homologous domains of these MMPs include the signal peptide domain, propeptide domain, catalytic domain and hinge region or linker peptide along with a hemopexin domain (4, 8). The hinge region connects the catalytic domain to the hemopexin domain (7, 9, 10). MMPs are divided into collagenases (e.g., MMP-1, MMP-8, MMP-13, and MMP-18) (11, 12), gelatinases (e.g., MMP-2 and MMP-9) (13, 14), stromelysins (e.g. MMP-3, MMP-10, and MMP-11) (15, 16), matrilysins (e.g. MMP-7 and MMP-26) (17), membrane-type MMPs (MT-MMPs) (MMP-14, MMP-15, MMP-16, MMP-17, MMP-24, and MMP-25), and others (MMP-12, MMP-19, MMP-21, and MMP-28) based on structural features and substrates (18–24). In particular, gelatinases have a special additional exosome insert in the catalytic domain called the collagen binding domain; matrilysins lack a C-terminal hemopexin-like domain linked by a hinge or linker region, and MT-MMPs have a C-terminal transmembrane domain with a short cytoplasmic tail (9, 25). The classifications and structures of MMPs are shown in **Figure 1**.

### 1.2 Regulation of MMPs at multiple levels

MMP activity is regulated in three different ways, through transcriptional regulation, inhibition by specific inhibitors, or activation by the proenzyme (26). At the transcriptional level, the activator protein (AP) -1 and -2 sites, the NF-κB site, the signal transducer and activator of transcription site, the polyomavirus enhancer-A binding protein-3 site, and others are key transcription binding sites for regulation of the *MMP* gene (8, 27–33). The AP-1 site is located close to the most proximal promoter of the typical TATA box and is the major mediator in *MMP* gene regulation. In many *MMP* promoters that contain AP-1 site, the juxtaposed transcription factor binding sites bind multiple erythroblastosis twenty-six factors, which determine the peculiarities among different genes and affect gene expression (8). The polyomavirus enhancer-A binding protein-3 site, can combine with members of the erythroblastosis twenty-six factors family of oncoproteins and work in synergy with the AP-1 site nearby to promote the production of MMPs among cancer cells for migration and invasiveness (28). MicroRNA is a non-coding single-stranded RNA. In liver cancer cells, mRNA expression of *MMP-2* and *MMP-9* is up-regulated as a result of the inhibition of miR-21, which stimulates the invasion and migration of tumor cells. miR-224 is associated with gene expression of *MMP-1*, which enables breast tumors to metastasize to the bone (34). NEMO-binding domain, a synthetic peptide corresponding to the S100A4-binding domain of methionine aminopeptidase 2 [MetAP2], blocks interaction between the metastasis−enhancing calcium−binding protein (S100A4) and its effector protein (MetAP2). And this blockage inhibits specificity protein 1 (Sp1) and ultimately leads to the downregulation of *MMP-14* gene expression (35). MMP-7 antisense oligonucleotides inhibit gene expression of *MMP-7* and inhibit the metastasis of gastric and colon cancer by interfering with protein translation or promoting mRNA degradation (36). The modulation of gene expression differs in various physiological and pathophysiological events, such as Ets1 enhancing gene expression of *MMP-1* through c-Jun (29, 30). Note that modulation of the *MMP* gene can be affected by several stimuli. Some factors, such as phorbol esters and ultraviolet B radiation, activate expression of the *MMP* gene, whereas others (37–39), such as transforming growth factor β (TGF-β), glucocorticoids and retinoic acid (40, 41), suppress it. In addition, the *MMP* gene may be induced indirectly by several signaling pathways. Inflammatory cytokines, for example, interleukin (IL)-1 and tumor necrosis factor, indirectly influence *MMP* gene expression and activate the ceramide signaling pathway. Three distinct MAP kinase pathways, p38, ERK1/2 and c-Jun N-terminal kinase (JNK) affect ceramide-dependent *MMP-1* gene expression in human skin fibroblasts (42–45). *MMPs* genes are not generally upregulated by gene amplification or mutation like classical oncogenes. Usually transcriptional changes and/or epigenetic modifications result in an upregulation of *MMP* gene expression in colorectal cancer (CRC) (46, 47). Besides transcriptional regulation, MMP activity is related to proMMPs, which are secreted as inactive zymogens. Extracellular activation of proMMPs involves two steps. First, the N-terminal sequence of the propeptide domain is cleaved and releases the Zn^2+^-binding site stemming from catalytic domain exposition. Second, propeptide cleavage resulting in an active form of enzyme (21, 48, 49). For example, proMMP-9 was activated and then generated MMP-9, thus catalyzing angiogenesis *via* the FGF-2/FGFR-2 pathway (50). In addition, the activity of MMPs is regulated by α2-macroglobulin and tissue inhibitors of MMPs (TIMs), the two main endogenous inhibitors (26, 51). Thiol-modifying reagents, sodium dodecyl sulfate, and oxygen radicals also induce activation of MMPs *in vitro*(52).Variation in temperature or a decrease pH in the physicochemical environment serves the same purpose (25).

### 1.3 Immunological function of MMPs

The immune system, human beings’ main defense against disease, is indispensable. It eliminates foreign invaders through the immune response in a sophisticated and scientific way. Components that participate in immune regulation include innate immune cells, which act as early-responders, and adaptive immune cells, which enhance the response and generate immunological memory and molecules. Cytokines and chemokines control the immunoreaction in time and space. They take part in cell migration to the site of inflammation, proliferation, intercellular communication, and cell death (53, 54). The immune response does not necessarily lead to an inflammatory response, but inflammation accompanies the immune response in most cases (55). Inflammation commonly occurs after infection and damage (56). When antigens enter the body, macrophages or epithelial cells secrete chemokines, causing an increase in the vascular epithelial cell gap and vascular expansion; a large number of neutrophils, mast cells, basophils, and eosinophils infiltrate from the blood vessels into tissue fluid, resulting in localized febrile response, redness, swelling, and pain the cardinal signs of inflammation. An excessive immune response can lead to inflammation, such as pathogenic microbial infection, tumor, autoimmune disease, and tissue damage induced by physical or chemical elements. Immune cells and cytokines play an important role in the occurrence and resolution of inflammation. Numerous immune cells (e.g., macrophages, neutrophils) infiltrate the inflamed area and activated immune cells to release inflammatory factors (e.g., TNF-α, IL-6 and IL-1β), which worsens the inflammation (56, 57). Resolving this inflammatory response requires the release of anti-inflammatory cytokines (e.g., IL-10) by immune cells (58–61).

The composition of the tumor microenvironment is very complex. The tumor stroma is composed of the ECM, immune cells, fibroblasts, endothelial cells and other non‐neoplastic cells (62). MMPs produced in immune cells take part in innate and acquired immunity (63). Many immune cells express low levels of MMPs in the resting state. In mouse splenic CD4+ T cells, membrane-anchored *disintegrin metalloproteinase-10 (ADAM-10)* and *ADAM-17* mRNAs are expressed highly, whereas the mRNA expressions of *MMPs*, such as *MMP-2*, *MMP-9* and *MMP-14* are low (64). Under normal conditions, when the expression of inflammatory cytokines and chemokines increases, MMPs of immune cells are secreted and activated. When stimulated by IL-8, TNF, or chemo-attractive formyl-Met-Leu-Phe peptide, MMP-9 in neutrophils is immediately released from gelatinase granules (also called tertiary granules) (65). In addition, TIMP1 protein is not expressed in neutrophils (66). However, B cells, T cells, and unstimulated human peripheral blood monocytes are able to express TIMPs in the steady state. The transcript levels of TIMP1, TIMP2 and TIMP4 are expressed more highly in monocytes than in B cells or T cells. In contrast, *TIMP3* mRNA is highly expressed in B cells (67). ADAM17 and MMP-8 cleave the lymphotoxin (LT)-α1β2 heterotrimer, causing the release of heterotrimers from polarized T helper 1 (TH1) and TH17 cells (68). The combination of LTα1β2 heterotrimers and LTβ receptor activates primary synovial fibroblasts, eventually leading to synovial inflammation (68). The OX40 (a member of the TNF superfamily)–OX40 ligand axis is involved in numerous inflammatory diseases, anti-tumor immune responses and metabolic syndromes (69). MMP-2-specific CD4(+) T cells exist in tumor-infiltrating lymphocytes from melanoma patients, and they have an inflammatory T(H)2 (Type 2 helper T cells) profile. Dendritic cells (DCs) with MMP-2 initiates TH2 responses against several melanoma-associated antigens. As a reaction to exogenous melanoma antigens, active MMP-2 promotes TH2 cell differentiation, degrades the IF-α/β receptor in immature DCs and increases the protein expression of OX40 ligand in mature DCs. Therefore, researchers speculate that the mechanisms by which activated MMP-2 promotes tumor development is as follows: MMP-2 polarizes tumor-infiltrating lymphocytes toward a TH2 cell phenotype, which restrains the tumoricidal TH1-type response. Moreover, MMP-2 inhibits the powerful promoter of TH1 cell polarization-IL-12 subunit p35 (IL-12α) (70).

Important factors in building an immune response are efficient migration of neutrophils along a chemotactic gradient and extravasation through blood vessels and tissues to sites of infection. MMPs play a role in these processes by modifying chemotactic agents. A cleavage mediated by MMP-7 releases the heparan sulfate proteoglycan syndecan 1, and its associated CXC-chemokine ligand 3 (CXCL3), which attracts neutrophils to the site of infection (71). MMP‐8 which is mainly produced by neutrophils can be detected in the inflammatory response and some malignant tumors. In one study, there was a persistent inflammatory response after MMP-8-deficient mice were injected with methylcholanthrene. The incidence of skin tumors in male mice of this type increased significantly; female mice that were treated with tamoxifen or had their ovaries removed were more likely than wild-type mice to develop tumors. These results indicate that MMP-8 has a tumor suppressor function to some extent (72). This function is also supported by the finding that MMP-8 inhibits melanoma growth *in vitro* and *in vivo* (73). MMP-8 is necessary for recruiting chemokine CXCL6 to activate neutrophils; neutrophils are not able to migrate to sites of LPS administration without MMP-8 (74). IL-8 is a prototype chemokine that activates neutrophils. There is positive feedback between MMP-9 and IL-8. Stimulated by IL-8, neutrophils secrete gelatinase granules whose main component is MMP-9 (65). MMP-9 truncates an amino-terminal fragment of IL-8 for large increases in IL-8 potency (75). MMP‐2 cooperates with MMP‐9 to promote neutrophil infiltration (76, 77). MMP-2 and MMP-9 have synergistic effects on cleaving CXCL5 to increase neutrophil migration to the peritoneum during IL-1β-induced peritonitis (78). Meprins are members of the metzincin superfamily of zinc metalloproteinases, the cleaved substrate involved in many pathological processes, such as inflammation, cancer and fibrosis. Meprins participate in activating MMP-9 in the immune response. MMP-3 is an efficient activator of proMMP-9. The cleavage mediated by meprins improves the activation kinetics of proMMP-9 by MMP-3 (79). In contrast, MMP-2 may suppress the inflammatory response inactivating monocyte chemotactic protein 3 or Chemokine (C-C motif) ligand 7 (80).

Monocyte precursors are capable of differentiating into local macrophages in tissues (81). In different microenvironments, the cell surface phenotypes and functions of macrophage populations are heterogeneous (82). Macrophages play a prominent role in anti-infection and, anti-tumor activity and immune regulation (83). Similar to what happens in neutrophils, macrophages move directionally along the concentration gradient of certain chemicals and accumulate at the site of the lesion, where these substances are released (56). Metalloproteinases are able to affect macrophage recruitment (84–86). In mice with TIMP3-null mammary glands, the inflammatory response is exacerbated, the number of CD3+ T-cells increases, and macrophage infiltration is more pronounced than in wild-type mice glands (87). A classic means of activating macrophages (classically activated macrophages or M1 macrophages) is through Toll-like receptor ligands and pro-inflammatory mediators, such as TNF-α, interferon-γ (IFN-γ), and IL-1. Additionally, alternatively-activated macrophages, or M2 macrophages, can also be alternatively activated by distinct mediators, like IL-4 and IL-13 (88, 89). In inflammation, M1 macrophages effectively dispose of infectious organisms, and orchestrate angiogenesis and the ingress of connective tissue cells to form a granuloma. The function of MMPs ECM remodeling is vital in that process (90). In healing, M2 macrophages may promote connective tissue cells to remodel the ECM (88, 89). *In vitro*-differentiated M1 macrophages, mRNA expression of *MMP-1, MMP-3, MMP-7, MMP-10, MMP-14 and MMP-25* are increased, and mRNA expression of *TIMP-3* is decreased. mRNA expression of *MMP-11, MMP-12, MMP-25* and *TIMP-3* are upregulated, whereas MMP-2, MMP-8 and MMP -19 were reduced in M2 macrophages (91). Researchers speculate that the function of macrophages is related to the profile of MMP expression profile. The upregulation of MMP-12 in M2 macrophages is a major influence on the formation of aneurysms (92). Higher levels of MMP-1 collagenase may might be linked to higher collagenolytic activity of M1 macrophages (91). Macrophages also participate in specific immune response by releasing either pro- or anti-inflammatory cytokines (93, 94). MMP-14 (MT1-MMP) can control inflammatory gene responses. MMP-14-deficient macrophages produce excessive chemokine and cytokine responses to immune stimulation both *in vitro* and *in vivo*: they increase the gene and protein expression levels of the pro-inflammatory *IL-12p40 *(also called *IL-12β*) and *IL-6*, along with decrease the gene and protein levels of the anti-inflammatory *IL-10*. Phosphoinositide 3-kinase δ (PI3Kδ), a key regulator of macrophage immune responses, is the downstream transcriptional target of MMP-14 (95–97). Protein expression of MMP-14-dependent PI3Kδ evokes the expression and activation of a PI3Kδ/Akt/GSK3β signaling axis, thus mediating the immunoregulatory Mi-2/nucleosome remodeling and nucleosome remodeling and deacetylase to limit the expression of proinflammatory mediators in macrophage (97–100). MMP-12 originating from macrophages participated in abrogating the acute immune response. In MMP-12-deficient mice, leukocytes accumulated at the site of infection. MMP-12 cleaves and inactivates numerous CXC-chemokines and CC-chemokines which are implicated in the influx of leukocytes at the site of inflammation (101).

MMP-7 is involved in the immune activity of macrophages and neutrophils. One immunological function of MMP-7 is proteolytically activating α-defensins (cryptdins), which are a group of six cationic anti-bacterial peptides that work by disrupting bacterial membranes (102, 103). When stimulated by bacterial products, such as LPS and lipoteichoic acid, α-defensins are secreted from neutrophils, monocyte/macrophages and Paneth cells at the base of the crypts in the small intestine (104). α-defensins also act as the chemo-attractants for monocytes, T-cells and DCs to connect innate immunity to adaptive immunity (104). α-defensins are also mitogenic for epithelial cells and fibroblasts to aid in wound healing (105).

The cDNA sequence of MMP-25 from Japanese sea bass (Lateolabrax japonicus) (LjMMP25) regulates the production of inflammatory cytokines and promotes phagocytosis and bactericidal activity in monocytes/macrophages. Moreover, LjMMP25 regulates the inflammatory response by modulating NF-κB activity during innate immunity (106). Macrophages have a negative impact on cancer treatment (107–109). They create an inflammatory environment to promote tumorigenesis and tumor progression, such as angiogenesis, migration and invasion, and immunosuppression (109). For example, the penetration of cancer cells and leukocytes into the cerebral vessels is a complex multi-step process. The activity of macrophage-derived MMP-2 and MMP-9 is pivotal to leukocyte’s ability to penetrate the parenchymal basement membrane in mice with the ability autoimmune encephalomyelitis. These MMPs can be inhibited to protect the brain parenchyma from damage by preventing the infiltration of leukocytes (110). Moreover, the activation of TNF‐α by MMPs contributes to tumor progression (63, 111). The membrane-bound precursor, proTNF‐α, is mainly expressed in macrophages. ADAM17 (a TNF-converting enzyme) and MMPs, such as MMP-1, MMP-2, MMP-3, MMP-9, MMP-12, MMP-14, MMP-15 and MMP-17, convert proTNF-α into TNF‐α (112).

## 2 MMPs in pathological processes

### 2.1 Multifaceted role of MMPs in biological and pathological processes

Under normal physiological conditions, the activities of MMPs are controlled by various stimuli at multiple levels. However, under pathological conditions, this dynamic balance is broken. Over-degradation of the ECM due to overactivation of MMPs, is associated with a great many diseases, such as cardiovascular disease (4, 113–115), arthritis (116), periodontal diseases (117–119), osteogenesis imperfecta (120), disorder of the central nervous system (121), tumor invasion and metastasis (34, 122), age-related macular degeneration (123) and many other pathological states (7). Moreover, a decrease in MMPs can give rise to hypertensive pregnancy, preeclampsia (124), and inflammatory damage (125, 126).

As mentioned above, there are several main types of MMPs including collagenases, gelatinases, stromelysins, matrilysins, and MT-MMPs. Different MMPs have different three-dimensional structures, along with corresponding specific inhibitors or drugs. A typical MMPs consists of a prodomain, a propeptide, a catalytic domain and a hemophosphate domain. Approximately 80 amino acids make up the propeptide domain, and about 170 amino acids make up the catalytic metalloproteinase domain. The polypeptide folding of the MMP catalytic domain is basically superposition. The domain consists of a five-stranded β fold sheet, three α helices, and connecting rings. It contains two zinc ions and up to three stable calcium ions. The joint peptide contained in MMPs consists of a hinge region of variable length and a hemophosphate domain of about 200 amino acids. Exceptions include MMP-7, MMP-26, and MMP-23, which lack the hinge region and heme domain; MMP-23 has unique cysteine-rich domains and immunoglobulin domains (127–129). MMP-1, MMP-8, MMP-13, and MMP-18 are collagenases, and their key feature is their ability to cleave interstitial collagen I, II, and III at specific sites three-fourths away from the N-terminus. MMP-2 and MMP-9 are gelatin enzymes, which have three type II fiber-fiber-domain repeats in the catalytic domain and can bind to gelatin, collagen and laminin to digest denatured collagen. MMP-3 and MMP-10 are matrix lysins that digest ECM components (21). The classification of common MMPs implied in pathological processes is shown in **Table 1**. Collagenases recognize the substrate *via* a hemopexin-like domain, degrade fibrillar collagen and affect the ECM environment (19). Collagenases are closely related to the occurrence and development of diseases characterized by the degradation or change in the ECM are closely related to collagenase, including heart failure, atherosclerosis, cancer, arthritis, abdominal aortic aneurysm (130–132, 136, 137, 167, 168). In addition, they play a protective role in some diseases, for instance hypertrophic cardiomyopathy (134). Furthermore, collagenases improve liver fibrosis (133, 138). MMP-1 inhibits the development of atherosclerosis (130). MMP-8 plays a protective role in arthritis (135). Another category of MMPs is gelatinase. These MMPs act as digestive agents for components of the ECM, such as type I and IV collagen (19). They are induced or inhibited by a diverse range of resolvable factors, including growth factors, cytokines and hormones, and are acted on by cellular contacts through specific signaling pathways (169). MMP-9 has pro-inflammatory properties, whereas MMP-2 has pro-homeostatic properties (169, 170). Gelatinases have a profound influence in inflammatory process and tumor progression and have therefore long been considered one of the most significant anti-tumor targets (139, 140, 171). In terms of non-neoplasticity, gelatinases are mainly involved in cardiovascular pathology and auto-immune diseases (20). Moreover, MMP-9 is associated with many respiratory diseases (143). A reduction in vascular MMP-2 and MMP-9 gives rise to hypertensive pregnancy and preeclampsia (124). Stromelysins, another class of MMPs, have a structural domain arranged similarly to that of collagenases. However, these MMPs do not cleave fibrillar collagen type I (19). An important physiological function of stromelysins is to activate other members of the MMP family (21, 129). The most widely described pathological role of stromelysins is in cancer progression (144, 147–149). In addition, they function in the progression of cardiovascular, degenerative, and auto-immune diseases (145, 146, 172). Matrilysins, yet another category of MMPs, do not contain a hemopexin-like domain and are able to decompose collagen type IV but not type I (19). Matrilysins are associated with a number of pathological conditions in humans, mainly cancer and, respiratory, cardiovascular, and neurological diseases (20, 143, 151, 153, 154). A large number of studies have demonstrated that MMP-7 acts in the development and migration of cancer (151, 173). Moreover, MMP-7 also has a critical role in pathogenesis of tonsillitis and permanent hearing loss (150, 152). A study confirmed the early role of MMP-26 in the invasion and angiogenesis of malignant tumors (139). The final member of the MMPs family discussed here is MT-MMPs, which are an important mediator of infiltration. The influence of MT-MMPs on pathological process is mainly reflected in their promotion of tumor invasion (140, 155–161, 165, 166). The ability to activate MMP-2 is one of the reasons why most MT-MMPs play these roles (173). A different example is MMP-17, which has no regulatory effect on MMP-2 although it still affects tumor invasion (174). In addition, studies have demonstrated that MT-MMPs are also implicated in the pathological process of osteoarthritis, atherosclerosis and Alzheimer’s disease (85, 132, 162–164). MMPs are medicinal targets highly relevant to the treatment of a variety of diseases. As understanding of the role of MMPs in biology and pathology increases, greater understanding of the structural similarities and differences among MMP families makes it possible to discover highly selective MMP inhibitors.

**Table 1:** The classification of common matrix metalloproteinases (MMPs) implied in pathological processes.

Category	Structural feature	MMPs	The related diseases	Effects and Mechanisms	Refs
Collagenases	Made by a pro-peptide, a highly Conservative N-terminal catalytic domain and a C-terminal hemopexin-like domain; The catalytic domain and the hemopexin-like domain are connected by an intermediate hinge region;	MMP-1	Atherosclerosis	Inhibit atherosclerosis development by degrading major constituents of vascular ECM and contributing to remodeling of ECM and destruction of plaque;	(130)
			Cardiac dysfunction	Produce loss of interstitial cardiac collagen in conjunction with a marked worsening of cardiac systolic and diastolic function;	(131)
			Osteoarthritis	Participate in matrix degradation in human articular cartilage, a phenomenon commonly observed in osteoarthritis;	(132)
			Liver fibrosis	Attenuate established fibrosis and induces hepatocyte proliferation;	(133)
			Hypertrophic cardiomyopathy	Promote degradation of collagen I and reduce passive diastolic dysfunction;	(134)
		MMP-8	Arthritis	Play a protective role in arthritis mice joints by reducing interleukin-1β, prokineticin receptor 2 and pentraxin-3 expression;	(135)
			Atherosclerosis	Increase degradation of interstitial collagen I, and contributes a “stable” fibrotic phenotype by causing serious intra-plaque hemorrhages;	(136)
		MMP-13	Atherosclerosis	Increase degradation of interstitial collagen I, and contributes a “stable” fibrotic phenotype by causing serious intra-plaque hemorrhages;	(136, 137)
			Osteoarthritis	Participate in matrix deterioration in joint cartilage in humans, commonly observed phenomenon in osteoarthritis;	(132)
			Liver fibrosis	Reduce collagen deposition and ameliorate liver fibrosis;	(138)
Gelatinases	Consists of a pre-peptide, a strongly reserved N-terminal catalytic domain and a C-terminal hemopexin-like domain; A curious additional lateral insertion in the catalytic domain, that is known as Collagen Binding Domain;	MMP-2	Colon adenocarcinoma	Enhance tumor invasion by discontinuing basement membrane deposition;	(139)
			Malignant gliomas	Involve in glial invasion and angiogenesis;	(140)
			Hypertrophic cardiomyopathy	Promote degradation of collagen I and reduce passive diastolic dysfunction;	(134)
		MMP-9	Colon adenocarcinoma	Enhance tumor invasion by discontinuing basement membrane deposition;	(139)
			Guillain-Barré syndrome	Promote to peripheral nerve dysfunction leading to demyelination in Guillain-Barré syndrome;	(141)
			Acute ischemic stroke	Associate with higher risk of death and severe disability;	(142)
			Malignant gliomas	Lie in remodeling associated with neovascularization;	(140)
			COVID-19 disease among obese-diabetic patients	Be associated with ARDS;	(143)
Stromelysins	Consists of a pre-peptide, a strongly reserved N-terminal catalytic domain and a C-terminal hemopexin-like domain;	MMP-3	Osteoarthritis	Participate in matrix deterioration in joint cartilage in humans, commonly observed phenomenon in osteoarthritis;	(132)
			Prostate cancer	Contribute to growth of prostate cancer in bone through intrinsic cell growth and extrinsic angiogenesis;	(144)
			Primary Biliary Cholangitis	Associate with liver function disorders and may play a role in the physiology of liver fibrosis in primary biliary cholangitis (PBC);	(145)
		MMP-10	Aortic stenosis	Play a core role in aortic stenosis calcification through phosphorylation of Akt;	(146)
			Non-small cell lung cancer	PKCiota-Par6alpha-Rac1 signaling axes actuates anchorage-independent proliferation and aggression of non-small cell lung cancer (NSCLC) cells through induction of MMP-10 production;	(147)
		MMP-11	Breast cancer	Enhance tumor cells migration and relate to poorly differentiated tumors;	(148)
			Colorectal cancer	Enhance tumor cells migration;	(149)
Matrilysins	Consists of a pre-peptide, a strongly reserved N-terminal catalytic domain; Lack a C-terminal hemopexin-like domain that is connected by a hinge or linker region;	MMP-7	Recurrent tonsillitis	Participate in recurrent tonsillitis progrssion by degrading ECM in response to inflammatory changes in tonsil tissue;	(150)
			Lung cancer	Cleave nucleolin which augment oncogenesis; Cleave the extracellular matrix and contributes to tumor aggression;	(151)
			Permanent hearing loss	Participate in modulating the cochlear reaction to sound hyperstimulation;	(152)
			Duchenne muscular dystrophy	Be related to duchenne muscular dystrophy cardiac dysfunction and myocardial fibrosis, possibly through remodeling of the extracellular matrix;	(153)
			COVID-19 disease among obese-diabetic patients	Be associated with ARDS;	(143)
			Multiple sclerosis	Promote the entry or restimulation of immune cells into the perivascular area, a key event in multiple sclerosis;	(154)
		MMP-26	Colon adenocarcinoma	Enhance tumor invasion by discontinuing basement membrane deposition;	(139)
MT-MMPs	Made by all protein domains specific to MMPs, from the N-terminal to the C-terminal (a propeptide, a catalytic domain, a hinge region and a C-terminal hemopexin domain); Have a C-terminal transmembrane domain that possesses a short cytoplasmic tail;	MMP-14 (MT1-MMP)	Cancer	Enhance tumor invasion by degrading ECM;	(155–158)
			Malignant gliomas	Involve in both glial invasion and angiogenesis;	(140)
			Osteoarthritis	Participate in matrix deterioration in joint cartilage in humans, commonly observed phenomenon in osteoarthritis;	(132)
		MMP-15 (MT2-MMP)	Gastric cancer	Enhance tumor invasion by degrading ECM;	(159)
		MMP-16 (MT3-MMP)	Meningiomas	Enhance tumor invasion;	(160)
		MMP-17 (MT4-MMP)	Atherosclerosis	Reduce atherosclerosis by inhibiting patrolling monocyte recruitment to early lesions;	(85)
			Breast cancer	Accelerates tumor proliferation, induces vascular enlargement and is associated with an increase in lung metastases;	(161)
		MMP-24 (MT5-MMP)	Alzheimer’s disease	Promote amyloid pathology associated with the ability of the protease to facilitate trafficking to one of the subcellular sites of amyloid production, cognitive decline, neuroinflammation and neuronal excitability;	(162–164)
			Breast cancer	Modulating cancer cell aggressiveness in a rigid ECM environment during tumor development;	(165)
		MMP-25 (MT6-MMP)	Colon carcinoma and brain tumor	Facilitate tumor development through its capacity to activate progressive proteinase A in the cell membrane;	(166)

In the tumor microenvironment, the activity of a variety of MMPs, including MMP-1, MMP-2, MMP-3, MMP-7, MMP-8, MMP-9, MMP-10, MMP-11 and MMP-14, are up-regulated. These MMPs participate in tumor proliferation, survival, and angiogenesis, enabling replication immortality, invasion/migration, immunity evasion and other processes (175, 176). These MMPs control tumor cell growth by the releasing of ectodomains of growth factor, regulating the bioavailability of growth factors and regulating signaling pathways related to cell proliferation (177). MMP-3 and MMP-7 expression in tumor cells may contribute to an apoptosis resistant phenotype (178, 179). In addition, the MMP family is necessary for tumor angiogenesis *via* a two-way action, that is promoting or inhibiting angiogenesis. MMP-1, MMP-2, MMP-7, MMP-9 and MMP-14 regulate this process, and the first three of them play critical roles (180–182). Another key process in which MMPs are the migration of tumor cells. MMP-14 is among the key contributors to cancer invasion and promotes cancer development by activating proMMP-2 and degrading the ECM to promote cancer migration (176). MMP-7 acts in tumor cell metastasis by activating the ERK 1/2 and JNK 1/2 signaling pathways (183). MMP-1, MMP-2, MMP-8, MMP-11, and MMP-13 are implicated in the regulation of tumor cell migration (184–188). Finally, MMPs, such as MMP-14, also participate in tumor immune monitoring (176, 189, 190). Increasing attention has been paid to the role of MMPs in tumor immune regulation, such as their effects on inflammatory and immune responses, the tumor immune microenvironment and their diagnostic or prognostic potential (191–196).

### 2.2 Relationship between MMPs and immune-related diseases

MMPs affect the process of colitis. MMP-2 is causative for inflammatory bowel disease (IBD), which is derived from weak mRNA expression of pro-inflammatory cytokines including *IFN-γ* and *TNF-α*, and weak protein expression of IL-6 and less overgrowth of the colonic lumen by potentially pro-inflammatory enterobacteria from the commensal gut microbiota (197). MMP-9 plays a potentially key role in the progress of ulcerative colitis (UC) by regulating the immune system (198). MMP-19 coordinates the appropriate innate immune response in colitis, which is critical to balancing the host response to colon pathogens (126). MMP-9 is a member of MMPs closely related to cancer. MMP-9 is related to immune infiltration in pan-cancer and can be used as a biomarker of cancer prognosis and metastasis (199). It is overexpressed in peripheral blood NK cells of prostate cancer (86). And MMP-9 also effectively reduces the tumor killing-effect of T cells *via* cutting the MHC class I molecule, cell surface antigen-presenting complex molecules expressed in melanoma cells (200). In addition, high expression of MMP-11 is associated with worse survival rate in breast cancer, which is related to a low immune response, such as the reduction in the number of CD8+T cells, CD4+T cells, B cells and activated DCs (201).

### 2.3 Relationship between MMPs and inflammatory diseases

Inflammation is a fundamental pathological process that occurs when biological tissue is stimulated by certain kinds of injury, such as trauma and infection. Topical presentations of inflammation include redness, swelling, heat, pain and functional impairment. Systemic reactions include fever and changes in peripheral blood levels. MMPs are vital elements implicated in the manifold regulation of inflammation (202, 203). In one study, levels of some MMPs, such as MMP-1, MMP-2, MMP-3, MMP-7, MMP-9, MMP-10, MMP-12, and MMP-13, were significantly elevated in ulcer biopsies from patients with inflammatory disease (204–208). MMPs have not only a negative influence (125), but they also have an impact on vascular permeability (209–211), ECM remodeling, epithelial proliferation, and angiogenesis in different stages of inflammation (4, 173). In a model of colonic injury induced by sodium dextran sulfate, MMP-10 had a positive effect on disease (172). Because the progression of damage due to lack of MMP-10 is accelerated with viciousness-potential, enhancing expression of MMP-10 is helpful (125). A similar observation can be found for MMP-19 (126).

## 3 Key MMPs in CAC and their immunomodulatory aspects

### 3.1 Important role of MMPs in colitis

Chronic inflammatory disease is often associated with the occurrence and development of various cancers. A classic example is the increased risk for CAC in patients with IBD. In chronic environments marked by chronic inflammation, the ECM is a major factor in maintaining and promoting tumor growth, and MMPs are the major protease involved in the pathogenesis of IBD. Although both sporadic CRC and CAC are malignancies of the colon, CAC differs from sporadic colon cancer in several respects. CRC is produced through three main pathways: the adenomato-carcinoma sequence, the serrated pathway, and the inflammatory pathway. In contrast, the development of CAC is associated with the inflammatory-dysplasia-carcinoma pathway. MMPs counteract ECM proteins expressed in the gastrointestinal tract during inflammation (212, 213). Therefore, this study was conducted to evaluate the role of MMPs in CAC and its related mechanisms (214). The essential role of these enzymes in the remodeling and destruction of tissue in IBD has been well documented (205, 215–220). Pathological results of IBD, progressive mucosal disintegration (e.g., ulcers and fistulas) and fibrosis due to excessive deposition of collagen (the main component of ECM), is related to a disruption in the balance between composition and breakdown of the ECM (221). As an important molecule in mucosa and submucosa, ECM is the substrate of MMPs, which is why MMPs play such an essential role in the development of IBD. In Crohn’s disease (CD), TNF-α and activated T cells stimulate mesenchymal cells to increase the secretion of MMPs, and then MMPs causes tissue damage by degrading the lamina propria matrix (222, 223). MMP-3 and MMP-9 participate in the formation of fistula in CD by degrading the ECM (224). Moreover, MMPs are the key element in wound healing in the late stage of IBD through their effects on degradation of the ECM (225). MMP-1, MMP-7, and MMP-10 are expressed in migratory enterocytes in this process (226), which is important for epithelial regeneration and wound granulation (225, 227, 228). Furthermore, MMP-3 is crucial in scar contraction and ECM remodeling (229–231).

Regarding the ECM, MMPs have roles in a diverse array of substrates (232), including cytokines (90, 233), chemokines (234–237), TNF-α (238), α1-antitrypsin/α1-antichymotrypsin (239), IL-1β (240, 241), stromal cell-derived factor-1 (234), growth factors (239) and so forth. Some factors, such as TNF-α and IL-1β, in turn, stimulate the production of MMPs (242–244). Injury to the intestinal barrier is also responsible for IBD. When the intestinal barrier is disrupted, gene and protein expression of *MMP-1*, *MMP-2*, *MMP-3*, *MMP-7*, *MMP-9*, *MMP-10* and *MMP-13* increase, and leukocytes are summoned to inflamed areas (219, 245–247). MMP-8 and MMP-9 are released from neutrophils to regulate proinflammatory cytokines and chemokines to increase the number of leukocytes and eliminate bacteria (204, 224, 245). Macrophages phagocytose bacteria, along with MMP-9 released externally and MMP-12 entering into the phagosome (248). MMP-12 has a direct bactericidal effect. Briefly, when bacterial pathogens invade, MMP-12 is mobilized to macrophage phagolysosomes and adhere to bacterial cell walls, destroying cell membranes and causing bacterial death (249). MMP-7 plays an indirect bactericidal role by activating and releasing bactericidal alpha defensins into the gut lumen (250, 251). MMP-10 from infiltrating myeloid cells participates in the recovery of DSS-induced damage to the colon (125). Research has also shown that the susceptibility to colitis, including significant disease progression, increased mortality, severe tissue destruction, increases level of pro-inflammatory regulators in the colon and plasma, and a significant delay in neutrophil infiltration and persistent inflammation, increased markedly in MMP-19-null mice. In IBD, MMP-14 in endothelial cells promotes angiogenesis, which is achieved by combining the C-terminal fragment of MMP-14 substrate thrombospondin-1 with CD47/αvβ3 integrin to produce nitric oxide (252). Moreover, the migration of macrophages that lack MMP-19 is reduced *in vivo* and *in vitro* and the mucosal barrier is damaged (126). Chemokine fractalkine (CX3CL1), a substrate of MMP-19, is an essential component of the response to DSS in acute colitis. Because CX3CL1 receptors exist on innate immune cells (e.g., macrophages, neutrophils), impaired immune cell trafficking may be associated with a lack of the soluble CX3CL1 in MMP-19-deficient mice. Mice without the receptor CX3CR1 have more serious symptoms of DSS-induced colitis (126, 253–255). The application value of MMPs as biomarkers in IBD has also been recognized. A number of studies have demonstrated the high sensitivity of MMP-9 in evaluation of active UC (256–258). In addition, through an analysis of emerging BiomARKers (EMBARK), the researcher not only proposed that the combination of fecal calprotectin and serum MMP-9 can be used as a biomarker of UC, but also confirmed the value of MMP-9 as a biomarker of CD, indicating the combination of fecal calprotectin, serum MMP-9 and serum IL-22 can be used as a biomarker of CD (259).

In conclusion, MMPs participate in the host immune defense, would healing, and epithelial regeneration and they have bidirectional effects in IBD. On the one hand, they are involved in the development of IBD through the process of inflammation. MMPs are indirectly associated with progressive organ damage, ulceration or over accumulation of collagen, the persistence of inflammation, and fibrosis because of their substrate ECM. On the other hand, some members of MMP family have an inhibitory effect on inflammation (215, 260).

### 3.2 Key MMPs in CAC

**Figure 2:**
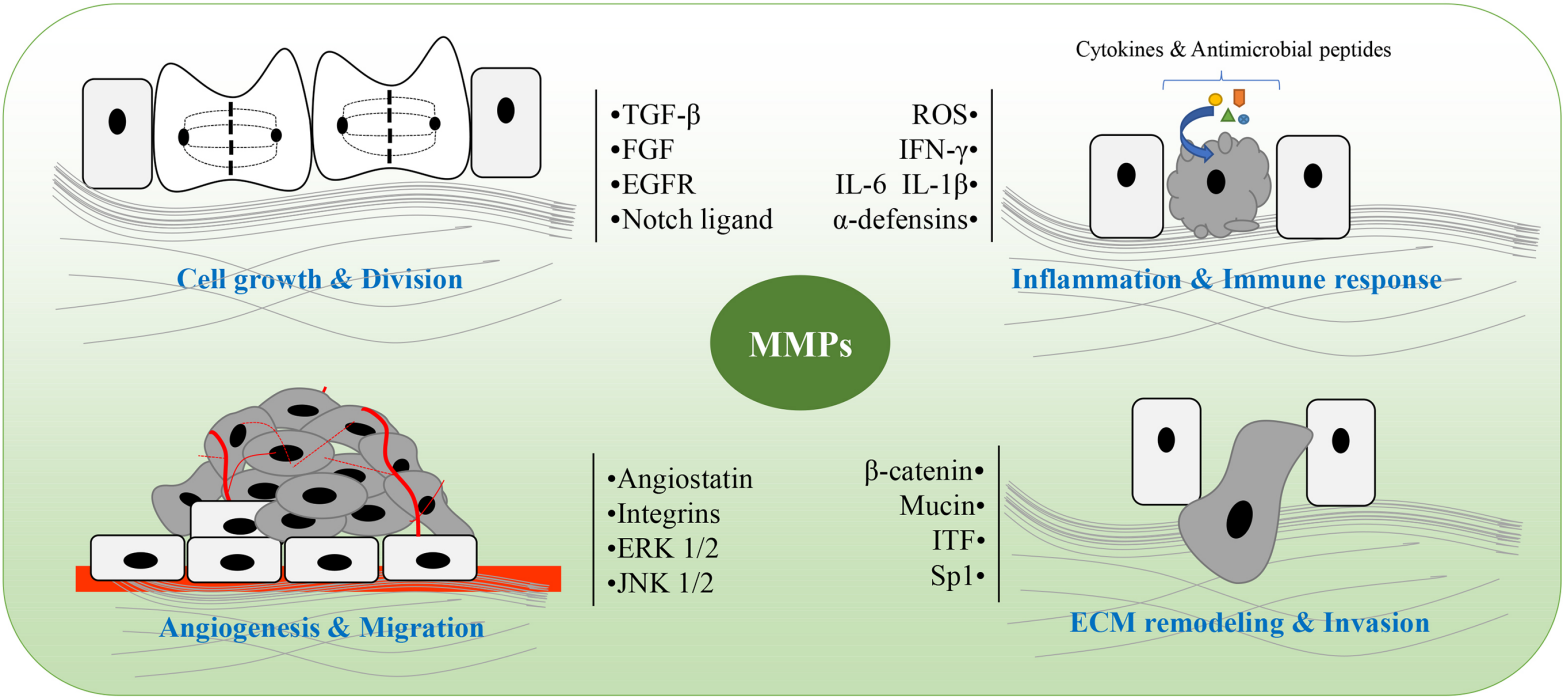
The main role of matrix metalloproteinases (MMPs) in colitis and colitis-associated cancers (CAC). MMPs are involved in pathological processes of colitis and CAC, including cell growth and division, angiogenesis and migration, ECM remodeling and invasion, as well as inflammation and immune response. ROS, reactive oxygen species; ITF, intestinal trefoil factor; Sp1, specificity protein 1; ECM, extracellular matrix.

**Figure 3:**
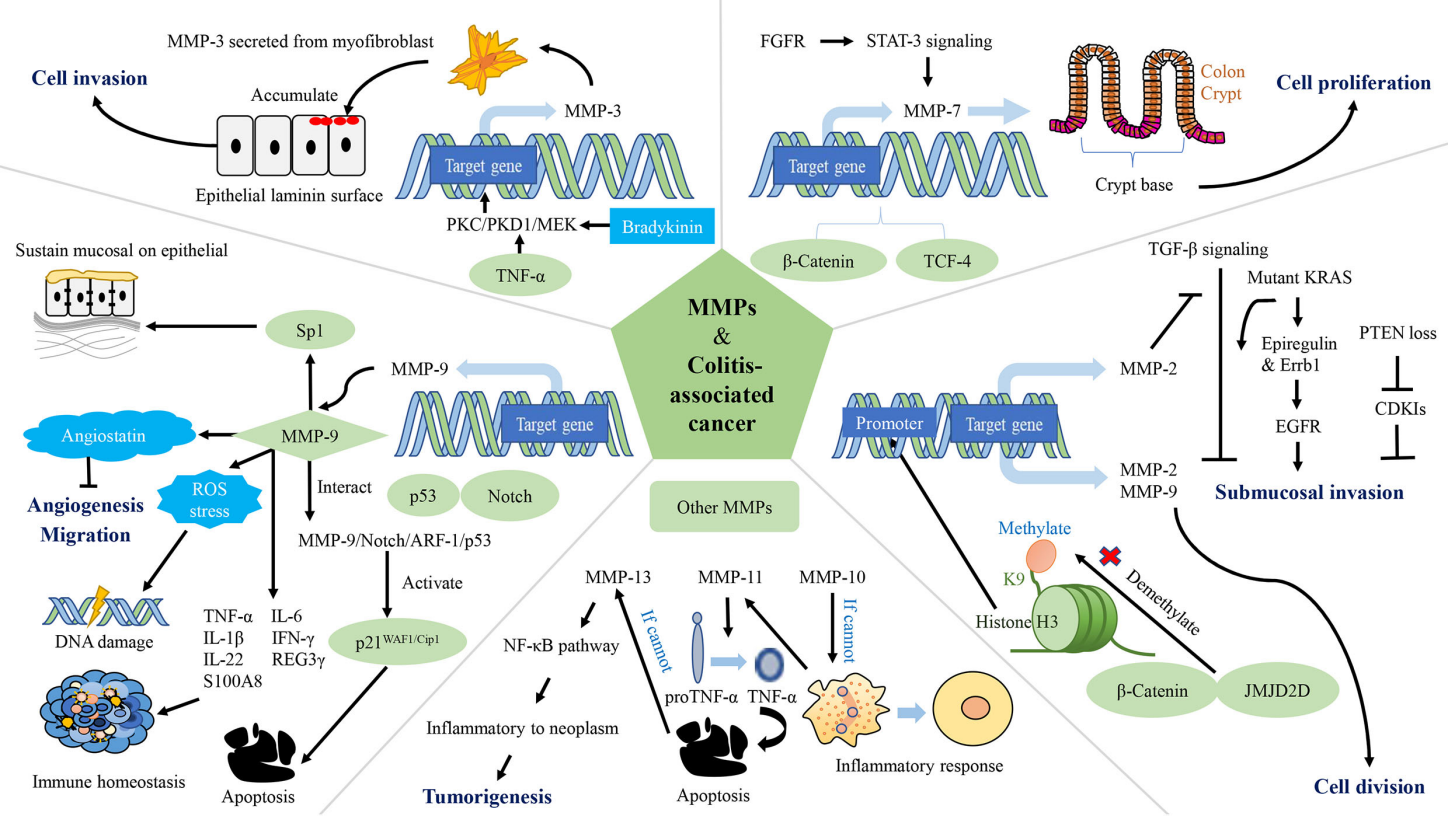
The network of matrix metalloproteinases (MMPs) interfering with colitis-associated cancers (CAC). MMP-2, MMP-3, MMP-7, MMP-9, MMP-10, MMP-11, and MMP-13 are involved in the regulation of CAC. MMP-2 and MMP-9 promote CRC cell proliferation. MMP-2 and MMP-3 contribute to tumor invasion. Expression of MMP-9 leads to apoptosis, reduced accumulation of ROS, DNA damage and inhibition of tumor vascularization, while maintaining epithelial mucosa, microbiota homeostasis and immune homeostasis. MMP-10 plays a role in inflammation regression. MMP-11 reduces apoptosis in cancer cells. PKC/PKD1/MEK: protein kinase C/protein kinase D1/mitogen-activated protein; TCF-4: T-cell factor-4; PTEN: phosphatase and tensin homolog deleted on chromosome 10; EGFR, epidermal growth factor receptor; FGFR, fibroblast growth factor receptors; CDK, cyclin dependent kinase; Sp1, specificity protein 1; ROS, reactive oxygen species.

CAC is a very common fatal complication of IBD (261–264). The pathogenesis of CAC is multifactorial, although a key driver of colitis is neoplastic progression (265–267). The lifetime risk for CAC in IBD patients is 15-40%, and CAC accounts for about 15% of mortality in these patients (268). Chronic inflammation generates oxidative stress that induces DNA damage that might activate some oncogenes and inactivate some anti-oncogenes (267). Related mechanisms include oxidative base lesions, replication stress, DNA crosslinking, and strand breaks, which eventually lead to genomic destabilization and tumorigenesis (269). MMPs play a roles in both promoting and inhibiting regulation of CAC development and progression, as shown in **Table 2**. The main role of MMPs in colitis and CAC is shown in **Figure 2**, and the network of MMPs that interfere with CAC is shown in **Figure 3**.** **A list of all genes mentioned here could be found in **Table S1**.

**Table 2:** The key matrix metalloproteinases (MMPs) in colitis associated cancer.

MMPs	Protein Expression	Molecular Mechanisms	Effects	Refs
MMP-2	↑	Histone demethylase (JMJD2D) and β-Catenin interacts physically (JMJD2D demethylates H3K9me3 on the promoter of β-Catenin target genes), hence this interaction increases promoter activity of target genes (including *MMP-2*) of β-Catenin, activates transcription of *MMP-2* and others; Macrophages infiltrate and express MT1-MMP, causing MMP-2 activation;	Promote CRC cell to proliferate, migrate and invade and form colorectal tumors in mice; Promote submucosal invasion of transforming growth factor (TGFB) signaling-repressed epithelial cells;	(270, 271)
MMP-3	↑	TNF-α and bradykinin enhance the expression of MMP-3 at a transcriptional level through protein kinase C /protein kinase D1 /mitogen-activated protein signal 20 pathway;	Promote tumor invasion;	(272–274)
MMP-7	↑	Lack of adenomatosis polyposis coli lead to deregulation of WNT signaling pathway, and binding accumulation of β-catenin and T-cell factor-4; Stat-3 signaling is activated by FGFR, thereby inducing MMP-7 expression;	Relate to the occurrence and development of CAC;	(275–278)
MMP-9	↑	Histone demethylase (JMJD2D) and β-Catenin interacts physically (JMJD2D demethylates H3K9me3 on the promoter of β-Catenin target genes), hence this interaction increases promoter activity of target genes (including *MMP-9*) of β-Catenin, activates transcription of *MMP-9* and others;	Activate p21^WAF1/Cip1^ by regulating notch activity, a key transcription factor in epithelial cell lineage, resulting in β-catenin inhibition and cell cycle arrest; Acts tumor suppressive effect by activating MMP-9-Notch1-ARF-p53 axis, which lead to apoptosis and DNA damage in colonic epithelium; Reduce reactive oxygen species accumulation and DNA destruction; Inhibit metastasis and adhesion of colorectal cancer cells; Reduce tumor angiogenesis; Act on EGFR-nuclear transcription factor-specificity protein 1 (Sp1) signaling pathway to sustain the epithelial mucosal and function as well as immune homeostasis; Maintain epithelial and mucosal integrity by increasing mucin and intestinal trefoil factor (ITF) and downregulating STAT3 pathway; Maintain the balance of microbiota;	(212, 213, 270, 276, 279–282)
MMP-10	↑	Activate proTNF-α turning into TNF-α, then promote NF-κB signaling pathway activation;	Destroy intestinal barrier function; Facilitate the resolution of inflammation;	(283)
MMP-11	↑	Associate with the increase of β-catenin accumulated crypts number;	Reduce apoptosis of cancer cells;	(276, 284, 285)
MMP-13	↑	Activate proTNF-α turning into TNF-α, then promote NF-κB signaling pathway activation;	Destroy intestinal barrier function; Facilitate the resolution of inflammation;	(283, 286)

MMPs degrade the protein components of the ECM and basement membranes, which provides a channel for cancer cells to invade to the vascular and lymphatic systems as well as promotes metastasis (276). In the process of tumor growth, MMPs are up-regulated, which strengthens the permeability of vascular endothelial cells, thereby increasing cell proliferation, migration and angiogenesis (287). Histone demethylase (JMJD2D) and β-catenin interacts physically (JMJD2D demethylates H3K9me3 on the promoter of β-catenin target genes), which increases the promoter activity of target genes (including *MMP-2* and *MMP-9*) of β-catenin; activates transcription of *MMP-2*, *MMP-9*, and others; and ultimately cause CRC cells to proliferate, migrate and invade, and form colorectal tumors in mice (270). In a mouse model of tumor invasion, macrophages infiltrate and express MT1-MMP, resulting in activation of MMP-2 and consequent inhibition of TGF-β. This process leads to submucosal invasion of epithelial cells when it occurs in conjunction with KRAS or phosphatase and tensin homolog deleted on chromosome 10 (271). Specifically, when the inhibition of TGF-β is accompanied by the expression of KRAS, activation of the epidermal growth factor receptor (EGFR) signaling pathway is increased as a result of increased protein expression of epiregulin and mRNA expression of *Errb1*. When the inhibition of TGF-β is accompanied by phosphatase and tensin homolog deleted on chromosome 10 deletion, mRNA expressions of *cyclin-dependent kinase (CDK) inhibitors (Cdkn2b/p15^Ink4b^*, *Cdkn1a/p21^Cip1^* and *Cdkn1b/p27^Kip1^)* is down-regulated (288, 289). The level of MMP-3 secreted from myofibroblasts is up-regulated in IBD and tumorigenesis (272, 273). TNF-α and bradykinin enhance expression of MMP-3 at a transcriptional level through the protein kinase C/protein kinase D1/mitogen-activated protein signaling pathway, and thus mediate CAC (272). This mediating effect is related to the promotion of tumor invasion by MMP-3 (274). MMP-7 is connected to the occurrence and progression of CAC, and is expressed intensely at crypt bases of epithelial cells and in dysplastic CAC biopsy, as observed in CRC (275). Because of the lack of adenomatosis polyposis coli, the WNT signaling pathway is deregulated and β-catenin and T-cell factor-4 accumulate. Hence, the expression of MMP-7 up-regulated (276, 277). In addition, fibroblast growth factor receptors in cancer-related fibroblasts activate Stat-3 signaling, thereby inducing MMP-7 expression (278). In contrast, the highly expressed MMP-9 in CAC inhibits the tumor by affecting the Notch signaling pathway. Specifically, MMP-9 activates p21^WAF1/Cip1^ by regulating notch activity, a key transcription factor in epithelial cell lineage, resulting in β-catenin inhibition and cell cycle arrest (213). MMP-9 from the colonic epithelium also acts as a tumor suppressor by activating the MMP-9-Notch1-ARF-p53 axis, which leads to apoptosis and DNA damage (279). Previous study claimed that epithelium-derived MMP-9 is beneficial for chronic inflammation, regardless of tissue origin, in contrast to neutrophil-derived MMP-9. They also proposed that MMP-9 (stemming from epithelium or neutrophils) is a pivotal regulator of acute IBD and sporadic cancers (279). MMP-9 reduces reactive oxygen species and DNA destruction in CAC as well (212). Some researchers have also found that the hemopexin domain of MMP-9 has an inhibitory effect on the metastasis and adhesion of CRC (280). The decrease in MMP-9 in plasma causes down-regulation of angiostatin synthesis, which results in tumor growth and vascularization (280, 281). MMP-9 expressed in the colonic epithelium maintains the microbiota balance. Antimicrobial peptides including REG3 and S100A families, are effective agents of the innate immune system (290–292). In transgenic mice constitutively expressing MMP-9 in the colonic epithelium mRNA levels of *TNF-α, IL-6, IL-1β* and *IFN-γ* increased, but mRNA levels of *IL-22, REG3γ* and *S100A8* decreased. MMP-9 maintains epithelial and mucosal integrity by increasing mucin and intestinal trefoil factor protein levels and down-regulating the STAT3 pathway *in vivo.* Moreover, MMP-9 acts on the EGFR-nuclear transcription factor-Sp1 signaling pathway to sustain epithelial mucosa and functioning as well as immune homeostasis (282).

MMP-9 and MMP-10 are only significantly expressed in inflamed tissue, not normal colon tissue, and they start to peter out when healing begins (276). MMP-10 is mostly expressed by macrophages. In UC, it is found in enterocytes at the margins of ulcers and in the cells of granulation tissue (276). Researchers believe that MMP-10 from infiltrating bone marrow cells plays a role in resolving the inflammation. With a lack of MMP-10, susceptibility to DSS-induced colitis increases, and prolonged IBD may eventually lead to dysplasia (276). In miR-148/152-deficient mice, expressions of MMP-10 and MMP-13 increases, thus activating pro-TNF-α turning into TNF-α and promotes activation of the NF-κB signaling pathway. Damaged functioning of the intestinal barrier accelerates colitis and CAC (283). Similarly, MMP-11 is virtually absent in regular tissues (276). The mRNA level of *MMP-11* is related to CAC in some way (284, 285). The mRNA level of *MMP-11* is up-regulated in CAC, and is associated with the increase in the number of β-catenin accumulated crypts (284). The proton pump inhibitor-omerprazole and TNF-α blocker-infliximab reduce the mRNA level of *MMP-11* and induces cells apoptosis in CAC (285). MMP-13 is highly increased in CAC colonic tissues, but do not change as the CAC progression (286). Compared to other MMPs, MMP-14 (MT1-MMP) does not increase markedly in CAC. Researchers have also found that Omerprazole and Infliximab were able to down-regulate the mRNA levels of *MMP-14 *(284). In a mouse model of CAC, miR-128, miR-134 and miR-330 are influenced by Dicer1. These microRNAs inhibit tumor growth *in vitro* and *in vivo* and modulate expression of MMP-3, MMP-10, and MMP-13 (285, 293).

### 3.3 Mechanisms underlying of typical MMPs in CAC

**Figure 4:**
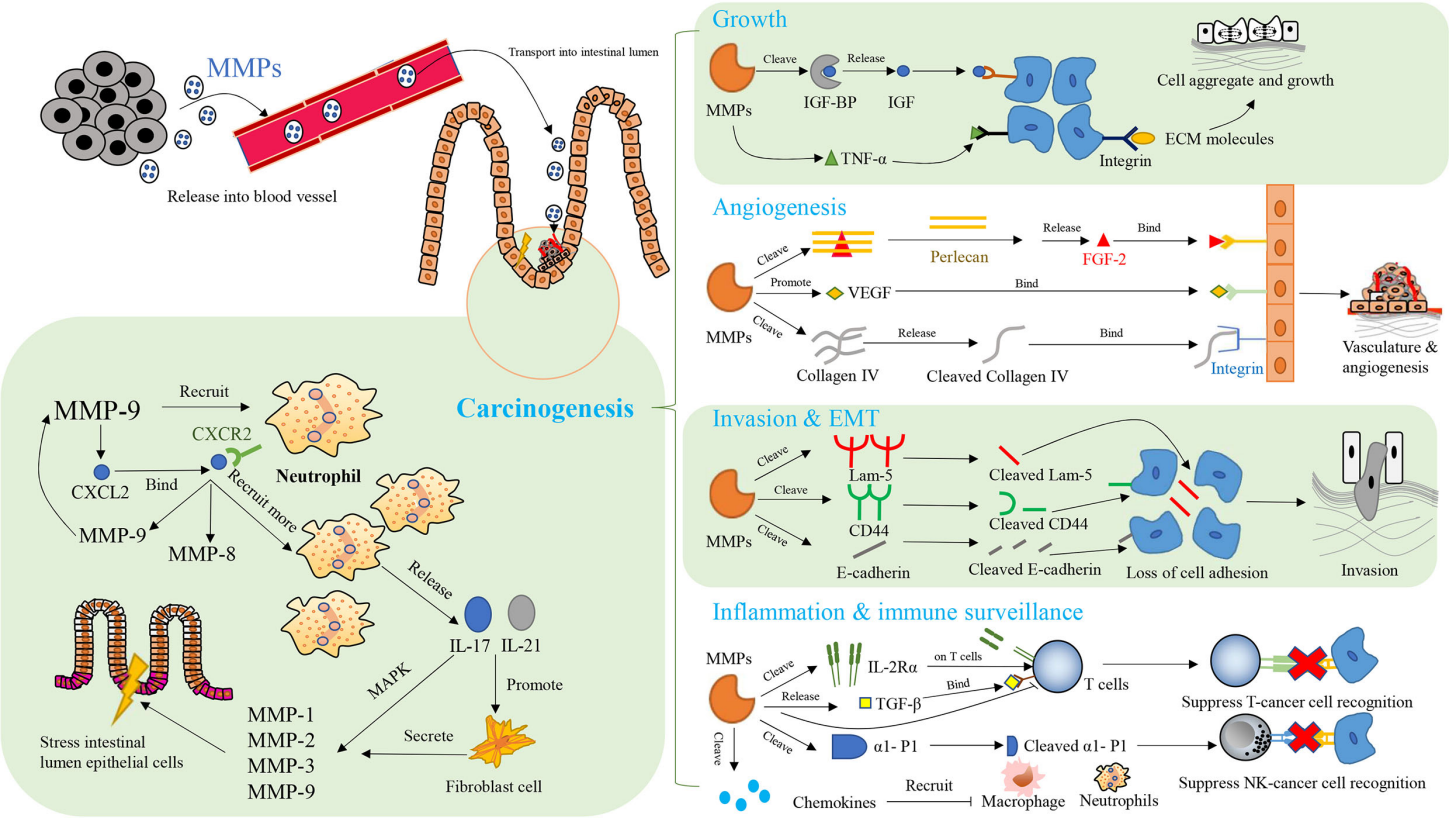
The network of MMPs and therapeutic targets in colitis-associated cancers (CAC). Key aspects of carcinogenesis are mediated by MMPs, including cell growth, angiogenesis, invasion, epithelial-mesenchymal transition (EMT), inflammation, and immune surveillance in immunomodulatory manner. CXCL2: CXC-chemokine ligand 2; CXCR 2: CXC chemokine receptor 2; MAPK, mitogen-activated protein kinases; FGF-2, fibroblast growth factor 2; IGF, insulin-like growth factors; IGF-BP, insulin-like growth factors binding protein; TNF-β, tumor necrosis factor-β; TNF-α, tumor necrosis factor-α; ECM, extracellular matrix; Lam-5, laminin-5; CD44, cluster of differentiation-44; VEGF, vascular endothelial-derived growth factor; α1-P1, alpha 1-proteinase inhibitor;IL-17, interleukin-17; IL-21, interleukin-21; IL-2Rα, interleukin-2 receptor alpha.

In CAC, typical MMPs affecting the organism’s immune function and their expressions are regulated by the immune system, as shown in **Figure 4** (10). MMP-7 decreases the sensitivity of mice to intestinal bacteria. Specifically, MMP-7 knockout mice do not activate pro-a-defensins in the gut to their mature active forms, with the result that these mice are highly susceptible to intestinal bacterial infection (250). MMP-8 affects the immune response to tumor and helps to resolve necrosis, which is positively related to the degree of primary tumor necrosis and blood neutrophil count, as well as negatively correlated with destructive inflammatory infiltration and Crohn’s-like lymphoid reaction (294). MMP-8, which is involved in resolving acute and chronic inflammation and helps to recruit neutrophils during acute inflammation, is mainly produced by neutrophils (295, 296). It plays a role in the recruitment of neutrophils to necrotic areas and in tissue remodeling, including collagen breakdown (294). MMP-9 is associated with the onset of lymphadenitis in patients with CAC, and is significantly up-regulated before the onset of lymphadenitis in these patients (297). In addition, MMP-9 maintains the integrity of epithelial mucosa and acts as a tumor suppressor in CAC, which is inseparable from its function of mediating the level of proinflammatory cytokines (282). The linings of gastrointestinal epithelial mucosa act as an external physical barrier and a functional immune barrier for an immune monitoring system (298). The imbalance in immune cells is crucial to the development of cancer (299). The inflammatory cytokines released by immune cells function in immune defense, and promote the development of cancer in specific circumstances (300, 301). MMP-9 increases mRNA levels of *IL-6, IL-1β, TNF-α* and *IFN-γ*, but decreases the mRNA level of *IL-22 *(282). Regarding the regulation of MMPs by the immune system, elevated MMP-8 is associated with systemic inflammation and increased secretion of various cytokines, including IL-1ra, IL-7 and IL-8, and is negatively associated with the number of tumors infiltrating mast cells (302). In a mouse model of colitis-associated CRC, the NF-κB mediated inflammatory reaction promotes protein expression of cyclin D1, phosphorylated ribosomal protein S6 and MMP-9 in the colon tissues of these mice, which plays a beneficial role in CRC progression (303). In the mouse model of CAC, MMP-9 expression is associated with excessive angiogenesis and cell proliferation, which is related to CXCL2 and neutrophil recruitment (304). CXCR2 is present in neutrophils and interacts with CXCL2 (305). This interaction promotes the recruitment of neutrophil and the synthesis of MMP-8 and MMP-9 (304, 306, 307). The proinflammatory factors IL-17 and IL-21 increase the MMPs secreted by human intestinal fibroblasts, including MMP-1, MMP-2, MMP-3 and MMP-9 (308–310). Among them, the inducing effect of IL-17 on MMP-1 and MMP-3 depends on the rapid activation of mitogen-activated protein kinase (308, 311). The regulation of MMPs by IL-21 does not occur at the level of transcription and translation and stimulating fibroblast with IL-21 does not increase the intracellular level of MMP RNA transcripts and proteins. The up-regulation of MMPs by IL-21 may depend on preferentially increasing the secretion of preconstituted or newly synthesized MMPs (309). In addition, expression of MMPs is regulated by TNF-α and IFN-γ (312).

### 3.4 Potential clinical applications of MMP inhibitors

Clinical trials of MMPs mainly focus on three factors. They are respectively the changes in clinical levels of MMPs in different disease states, the clinical use of MMP inhibitors in colitis and colorectal cancer, and combining MMPs and some regulatory factors with other drugs to control inflammation and tumors. MMP-2, MMP-3, MMP-7 and MMP-9 are the key MMPS in this process. However, current clinical trials have shown that inhibiting MMPs has no obvious effect on tumor responses, although it has a certain role in stabilizing the condition of diseases.

Many MMPs are upregulated in IBD. These MMPs remodel tissue and release several small protein fragments. In a clinical trial with 164 volunteers, these protein fragments could be used to distinguish between CD and UC. For example, measuring segments of vimentin (MMP-2 and MMP-8 decomposed and citrullinated-vimentin [VICM]) and type III (MMP-9 decomposed collagen type III [C3M]) can distinguish between CD and UC (313). A total of 138 participants took part in an IBD-related study, including different types of disease. Fecal MMP-9 can be used to diagnose and differentiate between UC and pouchitis, because it is strongly associated with clinical, histological, and endoscopic activities of different forms of IBD (257). A clinical trial evaluated the relationship between MMP and prognosis in CRC. This study enrolled 198 consecutive patients who had undergone operation for CRC, (85 females and 113 males). Of the patient, 67% were older than 65 years old, and their Tumor-Node-Metastasis classification ranged from 1 to 4. Expression of MMPs was higher in tumor tissue than in normal mucosa. This result indicates that high expression of MMP-2 and MMP-9 in the mucosa of CRC patients is related to poorer 5-year survival rates (314). MMP-7 is implicated in multiple processes of tumor development. To estimate the contribution of serum MMP-7 to the prognosis of resected CRC, researchers have conducted several clinical trials. In a study with 303 CRC patients (87 healthy controls, 96 nonmetastatic patients and 120 advanced patients), high serum MMP-7 was associated with a higher risk of death in terminal CRC patients (315). Included in another study were 175 curatively resected CRC patients. In two Cox proportional hazard models (overall survival and disease-free survival), higher MMP-7 was associated with higher recurrence and faster progression (316). Given the role of MMP-3 in cancer progression and metastasis, a study with 73 CRC patients who underwent minimally invasive colorectal resection investigated the relationship between increased plasma MMP-3 and residual metastases after surgery. Minimally invasive surgery directly up-regulated MMP-3 levels owing to surgery or subsequent wound healing or indirectly up-regulated MMP-3 by increasing TNF-α and IL-1 in the acute inflammatory response after surgery (317).

BAY 12-9566 inhibits MMP-2, MMP-3, and MMP-9. A phase I clinical enrolled 13 patients with colorectal, renal, gastroesophageal junction, duodenum, lung, and sarcoma cancer. Subjects were given BAY 12-9566 at four dosages. No tumor responses were found, but two patients had stable disease after 1.1 and 1.5 years of treatment (318). In another phase I clinical trial, 27 patients with advanced solid tumors took BAY 12-9566 100 to 1,600 mg/day. These patients had colorectal, lung, breast, ovarian and cervical cancers. The condition of 48% patients was stable. BAY 12-9566 did not reduce the size of the tumor, but slowed their growth (319). BMS-275291 is another wide-spectrum inhibitor of MMPs. In an open-label, phase I trial, 40 late-stage or metastatic cancer patients were given BMS-275291, most of them had CRC or non-small cell lung cancer. Although the researchers found no objective tumor responses, the condition of some patients stabilized (320).

Two clinical trials have been conducted on drug combinations. In a randomized, double-blind, clinical trial of rectal cancer, 34 patients receiving chemoradiotherapy were divided into a placebo group and a conjugated linoleic acid group. Supplementing conjugated linoleic acid decreased the levels of TNF-α, IL-1β, hsCRP, MMP-2 and MMP-9, which are biomarkers of tumor aggression and angiogenesis (321). A trial that included 37 patients with CRC lasted for 7 weeks. These patients who underwent chemotherapy, were separated into two groups: a fisetin group (n=18) and a placebo group (n=19). Flavonoid fisetin reduced levels of MMP-7, and significantly lowered levels of high-sensitivity C-reactive protein and IL-8 by the end of the study (322).

Despite the important role of MMPs in many human diseases, no broad-spectrum synthetic MMP inhibitor has successfully passed the clinical trial stage because of the bilateral pro-tumor and anti-tumorigenic effects of MMPs in cancer (323). A variety of MMPs, including MMP-2, MMP-9 and MMP-14, can degrade the basal layer of capillaries and promote exosmosis of tumor cells. MMP-9 also down-regulates the IL receptor on the surface of T cells, further inhibiting immunity and promoting cancer tolerance (324–326). By eliminating cell apoptosis, MMP-7 reduces the effect of chemotherapy even promoting tumor growth. However, MMP-8 may directly inhibit tumor metastasis in tumor cells. One of the side effects of broad-spectrum MMP inhibitors is that they interfere with the tumor-inhibiting function of MMP-8 (76). With more specific MMPs inhibitors now available, MMPs targeting can be reconsidered for cancer therapy (326).

## 4 Conclusion

Given their role in degrading the ECM, MMPs are associated with the occurrence and development of many diseases, especially inflammatory diseases. Most MMPs, such as MMP-2, MMP-3, MMP-7, MMP-9, MMP-10, MMP-12, and MMP-13 are increased in colitis and CAC. Therefore, reducing levels of these MMPs could effectively prevent the development of inflammation and CAC, as well as the progression of colitis-the eventual cause of CAC- from acute inflammation to chronic. However, the effect of some MMPs, like MMP-9, on CAC is bidirectional, which means they are involved in the pathogenesis of IBD and promote the metastasis and spread of malignant tumors, but also play a role in tumor suppression as well. Therefore, how to balance the bidirectional role of MMPs in clinical applications is a vital question. In specific diseases, it might be advisable to clarify the therapeutic target, especially the definitive role and efficacy of a certain MMP. Given their multifaceted role in colitis and CAC, more in-depth research is needed. In addition, MMPs participate in the host immune defense, wound healing, and epithelial regeneration. Normally MMPs are secreted and activated in immune cells when the expression of inflammatory cytokines and chemokines increase. MMPs modulate immune system activity by interfering with the differentiation and immune activity of immune cells, recruitment of macrophages, and migration of neutrophils. In clinical trials, the condition of CRC patients could be stabilized to a certain extent by inhibiting levels of MMPs. Therefore, levels of MMPs could be used to predict the condition and development of inflammatory diseases and CAC. Furthermore, MMPs have very broad prospects in the treatment of CAC through immunoregulation, which is also a promising direction in future research.

## Author contributions

LH and QK wrote and revised this manuscript. KC constructed figures in manuscript and helped to revise it. YZ helped to revise the manuscript and provided valuable feedback to this conception. ZZ revised the entire manuscript and edited the language for scientific presentation. The corresponding authors, ZZ and WT conceived and organized this study. All authors contributed to the article and approved the submitted version.
