# Supplementary material for: The immunomodulatory role of matrix metalloproteinases in colitis-associated cancer
Source: Front Immunol. 2023 Jan 19;13:1093990. doi: 10.3389/fimmu.2022.1093990 (PMC9910179; doi:10.3389/fimmu.2022.1093990)
Supplement: Supplementary file 1 [file Table_1.docx]

**The Immunomodulatory Role of Matrix Metalloproteinases in Colitis-Associated Cancer**

Luying He^1#^, Qianming Kang^1#^, Kalong Chan^2^, Yang Zhang^1^, Zhangfeng Zhong^2^*, Wen Tan^1^*

1. ^1^ School of Pharmacy, Lanzhou University, Lanzhou 730000, China

^2^ Macao Centre for Research and Development in Chinese Medicine, Institute of Chinese Medical Sciences, University of Macau, Macao SAR 999078, China

**RUNNING TITLE:** Immunomodulatory MMPs in CAC

**KEYWORDS:** Matrix metalloproteinases (MMPs), colitis associated cancer (CAC), Immunomodulation, inflammation, extracellular matrix (ECM)

^#^ The authors contributed equally to this work

* Corresponding authors

Dr. Zhangfeng Zhong, Macao Centre for Research and Development in Chinese Medicine, Institute of Chinese Medical Sciences, University of Macau, Macao SAR 999078, China. Phone: (+853) 88224676; Fax: (+853) 28841358. E-mail: zfzhong@um.edu.mo

Dr. Wen Tan, School of Pharmacy, Lanzhou University, Lanzhou, Gansu 730000, China. Phone: (+86) 0931 8915686; Fax: (+86) 0931 8915686. E-mail: tanwen@lzu.edu.cn

**Table S1. Formal gene symbol and description of the genes mentioned in the manuscript**

| Gene | Gene ID | Official full name | Gene type |
| --- | --- | --- | --- |
| *ADAM10* | 102 | ADAM metallopeptidase domain 10 | protein coding |
| *ADAM17* | 6868 | ADAM metallopeptidase domain 17 | protein coding |
| *Cdkn2b* | 1030 | cyclin dependent kinase inhibitor 2B | protein coding |
| *Cdkn1a* | 1026 | cyclin dependent kinase inhibitor 1A | protein coding |
| *Cdkn1b* | 1027 | cyclin dependent kinase inhibitor 1B | protein coding |
| *Errb1* | 13649 | epidermal growth factor receptor | protein coding |
| *IFN-γ* | 3458 | interferon gamma | protein coding |
| *IL-1β* | 5335 | interleukin 1 beta | protein coding |
| *IL-6* | 3569 | interleukin 6 | protein coding |
| *IL-10* | 3586 | interleukin 10 | protein coding |
| *IL-12β* | 3593 | interleukin 12B | protein coding |
| *IL-22* | 50616 | interleukin 22 | protein coding |
| *KRAS* | 3845 | KRAS proto-oncogene, GTPase | protein coding |
| *MMP1* | 4312 | matrix metallopeptidase 1 | protein coding |
| *MMP2* | 4313 | matrix metallopeptidase 2 | protein coding |
| *MMP3* | 4314 | matrix metallopeptidase 3 | protein coding |
| *MMP7* | 4316 | matrix metallopeptidase 7 | protein coding |
| *MMP8* | 4317 | matrix metallopeptidase 8 | protein coding |
| *MMP9* | 4318 | matrix metallopeptidase 9 | protein coding |
| *MMP10* | 4319 | matrix metallopeptidase 10 | protein coding |
| *MMP11* | 4320 | matrix metallopeptidase 11 | protein coding |
| *MMP12* | 4321 | matrix metallopeptidase 12 | protein coding |
| *MMP13* | 4322 | matrix metallopeptidase 13 | protein coding |
| *MMP14* | 4323 | matrix metallopeptidase 14 | protein coding |
| *MMP19* | 4327 | matrix metallopeptidase 19 | protein coding |
| *MMP25* | 64386 | matrix metallopeptidase 25 | protein coding |
| *PTEN* | 5728 | phosphatase and tensin homolog | protein coding |
| *REG3-g* | 19695 | regenerating islet-derived 3 gamma | protein coding |
| *S100A8* | 6279 | S100 calcium binding protein A8 | protein coding |
| *TIMP1* | 7076 | TIMP metallopeptidase inhibitor 1 | protein coding |
| *TIMP2* | 7077 | TIMP metallopeptidase inhibitor 2 | protein coding |
| *TIMP3* | 7078 | TIMP metallopeptidase inhibitor 3 | protein coding |
| *TIMP4* | 7079 | TIMP metallopeptidase inhibitor 4 | protein coding |
| *TNF* | 7124 | tumor necrosis factor | protein coding |
| *TXNIP* | 10628 | thioredoxin interacting protein | protein coding |

*Note: References from https://www.ncbi.nlm.nih.gov/gene/*
